# Fluorescence-guided inguinal hernia repair with heightened nerve visualization to prevent chronic post-operative inguinal pain: Case report

**DOI:** 10.1016/j.ijscr.2025.110911

**Published:** 2025-01-20

**Authors:** Fernando Dip, Jorge Luis Harraca, Alberto Rancati, Diego Sinagra, Raul J. Rosenthal

**Affiliations:** a Hospital de Clínicas José de San Martín, Buenos Aires, Argentina; b Hospital Privado de Rosario, Rosario, Santa Fe, Argentina; c Sanatorio Otamendi, Azcuénaga 870, CABA C1115AAB, Argentina; d Cleveland Clinic Florida, Weston, FL, USA

**Keywords:** Case report, Inguinal hernia repair, Iatrogenic nerve injury, Nerve-sparing surgery, Fluorescence imaging, Dendrite® imaging system

## Abstract

**Introduction:**

Iatrogenic injury to the ilioinguinal nerve and its branches during anterior inguinal hernia repair is a cause of chronic inguinal pain in up to 12 % of patients undergoing this operation. The risk of nerve injury is high, given the nerves' relatively small caliber and strictly-confined space through which they pass. In the current report, we describe using a novel fluorescence imaging system developed to detect nerve autofluorescence in a 66-year-old man who presented with a left-sided Type II inguinal hernia and underwent inguinal hernioplasty.

**Case presentation:**

Under general anesthesia, a left inguinal hernioplasty with mesh was performed using the Lichtenstein technique through an anterior approach. During surgery, a Dendrite® Imaging camera (Dendrite® Imaging, Germany) was employed to allow the surgical team to alternate freely between standard operating room (white) light and near-ultraviolet light (NUVL), specifically to enhance visualization of the ilioinguinal nerve and its branches. Under white light, neither the ilioinguinal nerve nor any of its branches were clearly visible. However, under NUVL, all fluoresced brightly and were easily avoided throughout the course of the hernia repair. The operation proceeded with no intraoperative or postoperative complications.

**Discussion:**

In this case, autofluorescence of the ilioinguinal nerve and its branches under NUVL utilizing a novel, hand-held fluorescent camera during hernia repair aided in their visualization and appeared to help prevent nerve injury.

**Conclusion:**

New intraoperative technology that allows nerves to auto-fluoresce intra-operatively under NUVL warrants larger series and comparative trials to evaluate its efficacy at reducing iatrogenic nerve injury during inguinal hernioplasties.

## Highlights

• Iatrogenic injury to the ilioinguinal nerve is a common source of chronic post-operative pain after inguinal hernia repair
• Using novel imaging technology, nerves auto-fluoresce in near-ultraviolet light
• During inguinal hernia repair, using the Dendrite® imaging system facilitated visualization of all nerves in the surgical field.
• Iatrogenic nerve injury was avoided.

## 1 Introduction

Inguinal hernia repair is a widely performed surgical procedure, performed on >20 million people annually and considered the most frequently performed operation in the world [1]. Such numbers make any common complication resulting from this procedure of enormous healthcare and societal impact.

In the past, the most significant long-term adverse event was hernia recurrence, with an incidence rate of up to 15 % that appears to have decreased significantly since the advent of prostheses like a polypropylene mesh [2,3]. However, although recurrences no longer seem to be the concern they once were, chronic postoperative inguinal pain, stemming from intra-operative, iatrogenic injury to the inguinal nerve and/or one or more of its branches, has now emerged as a prominent issue spawning considerable clinical and scientific debate. Several studies have reported incidence rates for chronic inguinal pain after hernia repair as high as 20 %, which can transform patients' surgical experience into a highly negative one, affecting both their social and work life and generating numerous legal claims against surgeons [4,5].

Neuropathic pain can be caused by various neuroanatomical sources of direct or indirect injury to the inguinal nerve and its branches, making their visualization during the surgical procedure critically important. Various American and European guidelines for managing inguinal hernias have considered this topic, and they recommend focusing on the localization and visualization of the inguinal nerve and its branches to avoid their compromise and reduce the incidence of postoperative pain [1,6]. Such visualization can be difficult, however, given these nerves' relatively small caliber and the tightly-confirmed space through which they travel. Securing the mesh commonly utilized to prevent hernia recurrence also requires the placement of several sutures, any one of which can transect a poorly-visualized nerve. There also is the risk of stretch injury as herniated tissue is mobilized back into proper alignment.

To follow current guidelines emphasizing the cruciality of nerve visualization, we have started to employ a novel fluorescence imaging system (Dendrite® Imaging, Germany) developed in recent years that makes nerves auto-fluoresce when viewed in near-ultraviolet light (NUVL) [7]. Its use has been documented *in vivo* in both Wistar rats, where the nerves are generally very small [8], and in patients undergoing a wide range of surgical procedures, including parotidectomies, thyroidectomies, and spinal and peripheral nerve surgeries [9]. More recently, this imaging system has been found to be 100 % sensitive and 100 % specific for nerves, identifying all 81 recurrent laryngeal nerves sought in 65 patients undergoing a thyroidectomy [10].

Here, we describe our use of this innovative imaging system to visualize the inguinal nerves and their branches within the surgical field and prevent their iatrogenic injury, thus avoiding the serious complication of chronic neuropathic pain following inguinal hernia repair.

## 2 Case presentation

This case report has been drafted in full compliance with 2023 SCARE guidelines [11]. A 66-year-old man with grade 1 obesity and no medical or surgical history consulted electively for a left inguinal region mass of four years evolution with progressive growth over the last year. On physical examination, a left indirect inguinal hernia was found that was both reducible and coercible, and thereby compatible with Type II in the modified Gilbert classification system. The hernia was not painful, either spontaneously or on palpation. An anterior inguinal hernioplasty with polypropylene mesh was scheduled.

**Fig. 1:**
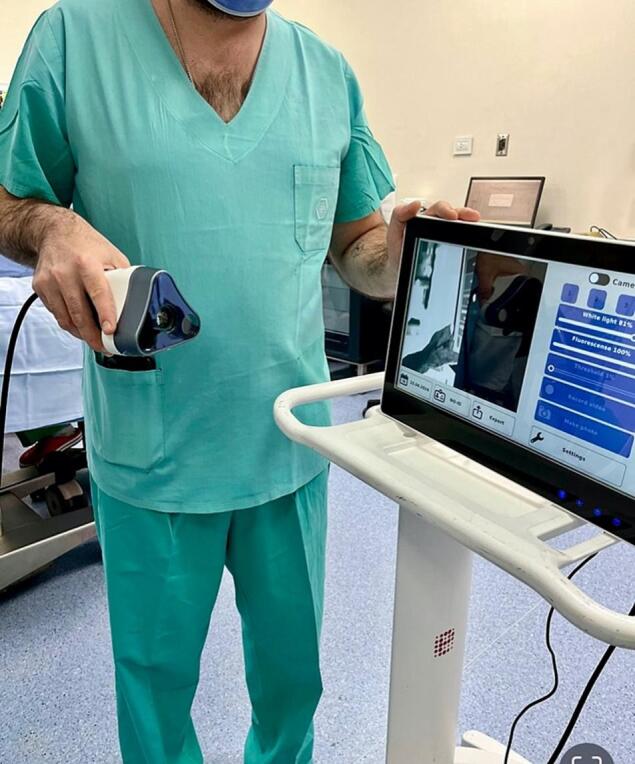
Dendrite® Imaging system. The Dendrite® Imaging system employs a near-ultraviolet light (NUVL) source and filter system to detect fluorescent signals emitted by neural tissue. These signals then pass through a filter system within the camera head, are captured by a chip, and are transmitted to a dedicated software platform for real-time analysis, processing, and relay to a display screen, allowing the surgical team to observe neural structures clearly fluorescing.

The operation was performed using the Dendrite® Imaging system to permit visualization of the surgical field under both standard operating room (white) light and NUVL (Fig. 1.). For the surgery, the patient was placed in a supine position, and a left inguinal incision was made. Standard operating procedures were followed, except for using the Dendrite camera, which allowed the surgeon to alternate freely between standard operating room light and NUVL, as needed. Under white light, neither the ilioinguinal nerve nor any of its branches were clearly visible. However, under NUVL, all structures glowed intensely, allowing the surgical team to visualize all critical nerve structures, including the ilioinguinal nerve trunk and its branches.

**Fig. 2:**
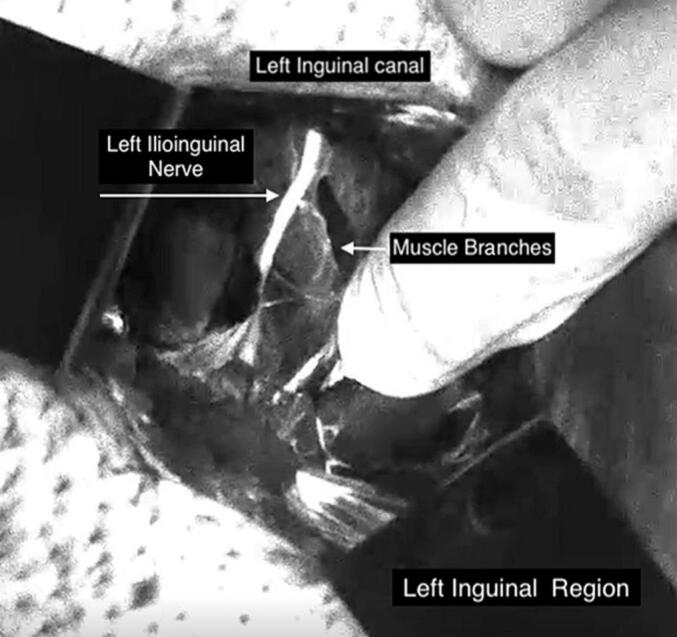
Surgical field in near-ultraviolet light, revealing autofluorescence of the left ilioinguinal nerve (LIN) and its muscular branches. In white light, the LIN was difficult to distinguish from background tissue and none of its branches were visible.

Dissection of the anatomical planes of the left inguinal region was performed, exposing the inguinal canal without dissecting the nerve to preserve nerve integrity and visualizing it through autofluorescence using the camera. The hernial sac was treated after reducing its contents, and repair was performed using the Lichtenstein technique with a polypropylene mesh, fixed with polypropylene sutures, thereby avoiding direct contact with the ilioinguinal and other nerves previously identified under NUVL at fixing points in the muscular plane (Fig. 2). Anatomical planes then were closed. The entire procedure took 45 min. Post-operatively, the patient had a good course, being discharged 6 h after the procedure. All post-operative analgesics were discontinued within 48 h of surgery, with no pain recurrence. He remains asymptomatic eight months after surgery, having returned to performing all his usual activities.

## 3 Discussion

Inguinal hernia repair can cause significant complications. Among them, post-herniorrhaphy neuralgia is a potentially disabling condition. The most common cause of nerve injury is failure to identify and protect nerves intra-operatively. To reduce the incidence of this disabling complication of hernia surgery, the surgeon must have a complete understanding of inguinal anatomy and avoid both direct and indirect trauma to the ilioinguinal nerve and its branches.

Various guidelines for managing inguinal hernias in 2018 and an update in 2023 have issued strong recommendations for surgeons to learn and recognize nerve anatomy during surgery to reduce the incidence of chronic post-herniorrhaphy pain [1,6]. Sometimes exaggerated dissection while searching for the ilioinguinal nerve results in its damage, highlighting the importance of recognizing the nerve as early in the procedure as possible.

In the quest to recognize the nerve without dissection, we considered that autofluorescence has been documented in other critical anatomical structures, notably the parathyroid glands, which are particularly susceptible to iatrogenic injuries during thyroidectomy procedures. In a randomized clinical trial comparing parathyroid gland visualization during thyroidectomy using only standard operating room (white) light *versus* adding a near-infrared light (NIRL) fluorescence imaging system, switching from white light to NIRL increased the number of glands detected from 2.6 to 3.5 per patient (*p* < 0.001), revealed at least one gland not visible with white light in 67.1 % of patients, and reduced the rate of postoperative hypocalcemia requiring treatment tenfold (*p* = 0.005) [12].

However, nerves do not auto-fluoresce with NIRL. Nor is their visualization generally improved using fluorescent dyes like indocyanine green, except for large nerves with a highly vascularized perineurium. Initially *in vitro*, then *in vivo* in Wistar rats [8], and more recently *in vivo* in human patients during surgery, evidence has accumulated that nerves auto-fluoresce when viewed under NUVL using a recently-developed nerve imaging system [9,10]. In a study published in 2024 involving 65 patients undergoing thyroidectomy, in which the fluorescence intensity of the recurrent laryngeal nerve (RLN), thyroid gland, and other background tissues was measured using ImageJ software, all 81 RLNs sought (16 bilateral resections) were clearly distinguished from the thyroid and all other background tissues, with mean relative fluorescence unit (RFU) scores of 134.3, 33.7, and 14.4 (*p* < 0.001), respectively [10]. Additionally, under NUVL, 76 % more RLNs and more than twice the RLN branches were observed compared to white light, and the average length of the nerve visualized was 32 % greater (average nerve lengths = 2.5 *vs.* 1.9 cm, respectively), all differences statistically significant at *p* < 0.001. Nerves up to 1 mm wide were visualized under NUVL. And the lowest RFU count recorded for any of the 81 RLNs visualized under NUVL was higher than the highest RFU count recorded for any of the background tissues.

Based on these observations and evidence that nerves auto-fluoresce when viewed under NUVL, use of the Dendrite camera in the current case was fundamental to clearly localizing the nerves and following them throughout their course in the surgical field. This included the ilioinguinal nerve and its small branches, consistent with the 1 mm wide nerves detected during thyroidectomies [10]. Additionally, this technology allowed the hernioplasty surgical team to perform a meticulous nerve-preserving technique that prevented neurological damage and its postoperative symptoms. Given that up to 12 % of patients have been reported to suffer chronic inguinal pain following anterior-approach inguinal hernioplasty, we suggest that this technology should be tested more extensively, with specific comparisons against white light, to validate its ability to reduce this complication.

One limitation of the current technology is that, at the current time, the camera can only be used during open surgeries. However, at this time, open remain more common than laparoscopic hernia repairs worldwide, and in a 2023 retrospective analysis of 106,529 patients in the US Veterans Affairs database who underwent surgery from 1998 to 2019, though laparoscopic procedures took longer to complete, there were no significant differences in any patient outcomes [13]. Nonetheless, both integration of the current near-ultraviolet light technology into a laparoscope and rigorous study of this next step's effectiveness certainly seem warranted.

## 4 Conclusion

Fluorescence imaging that allows nerves to auto-fluoresce warrants a larger series of patients and comparative trials to evaluate its efficacy at reducing iatrogenic nerve injury during inguinal hernioplasties.

## Author contribution

NR was the main author of the paper. However, all the authors contributed to study design and paper writing, and all edited the manuscript prior to submission.

## Informed consent

Written informed consent was obtained from the patient for publication and any accompanying images. A copy of the written consent is available for review by the Editor-in-Chief of this journal on request. Anonymity has been ensured throughout the publication process.

## Ethical approval

Since this was standard surgical care, the ethics committee at Hospital Privado de Rosario informed us that no ethics application was required. All patients provided informed written consent.

## Guarantor

Dr. Ingenito is the study guarantor.

## Research registration number

This is not the first time that Dendrite camera use has been reported for a human patient.

## Funding

This study received no funding from any external source.

## Patient's perspective

The patient remained very satisfied with the intervention and expressed no complaints, returning to his usual activities without discomfort.

## Conflict of interest statement

Dr. Dip is a stakeholder in Dendrite® Imaging (Germany) and a member of Dendrite's Imaging's board of directors. Dr. Rancati is a member of Dendrite® Imaging's Board of Directors. Dr. Harraca is a member of Dendrite® Imaging's advisory board. Neither Dr. White nor Dr. Sinagra have any conflicts of interest.
